# Hospital Trusts productivity in the English NHS: Uncovering possible drivers of productivity variations

**DOI:** 10.1371/journal.pone.0182253

**Published:** 2017-08-02

**Authors:** María Jose Aragon Aragon, Adriana Castelli, James Gaughan

**Affiliations:** Centre for Health Economics, University of York, Heslington, YORK, United Kingdom; Azienda Ospedaliero Universitaria Careggi, ITALY

## Abstract

**Background:**

Health care systems in OECD countries are increasingly facing economic challenges and funding pressures. These normally demand interventions (political, financial and organisational) aimed at improving the efficiency of the health system as a whole and its single components. In 2009, the English NHS Chief Executive, Sir David Nicholson, warned that a potential funding gap of £20 billion should be met by extensive efficiency savings by March 2015. Our study investigates possible drivers of differential Trust performance (productivity) for the financial years 2010/11-2012/13.

**Methods:**

Following accounting practice, we define Productivity as the ratio of Outputs over Inputs. We analyse variation in both Total Factor and Labour Productivity using ordinary least squares regressions. We explicitly included in our analysis factors of differential performance highlighted in the Nicholson challenge as the sources were the efficiency savings should come from. Explanatory variables include efficiency in resource use measures, Trust and patient characteristics, and quality of care.

**Results:**

We find that larger Trusts and Foundation Trusts are associated with lower productivity, as are those treating a greater proportion of both older and/or younger patients. Surprisingly treating more patients in their last year of life is associated with higher Labour Productivity.

## Introduction

Health care systems in OECD countries are increasingly facing economic challenges and funding pressures, which normally translate into interventions (political, financial and organisational) aimed at improving the efficiency of the health system as a whole and its single components, i.e. hospitals [1].

In 2009, the English NHS Chief Executive, Sir David Nicholson, warned that the English NHS, due to financial pressures faced by the UK government, needed to meet a potential funding gap of up to £20 billion. The funding gap–the so-called Quality, Innovation, Productivity and Prevention (QIPP) challenge, also known as the ‘Nicholson challenge’–was expected to be met by extensive efficiency savings by March 2015. Nicholson suggested such efficiency savings should be achieved through nationally-driven changes such as pay restraint (40%); improved efficiency in hospitals and other health services (40%); and transforming how services are delivered, e.g. treating more patients as day care cases rather than as overnight stays, (20%) [2–4].

In this policy and financial environment, optimising productivity is all the more vital. Variation in productivity can indicate the presence of unnecessary additional cost at one end of the spectrum and innovative best practice at the other. The aim of our study is to identify possible drivers of differential hospital productivity for the years immediately following the announcement of the Nicholson challenge, 2010/11–2012/13.

Appleby et al.’s [2] report includes a qualitative study of six providers’ attempts to improve productivity. Approaches noted include changes to infrastructure (including use of I.T.), workforce (including skill-mix) and clinical practice (including reducing length of stay and moving care to less intensive settings). This is presented within a larger review of the national picture. A report by the Health Foundation [5] focuses on changes in Trusts’ productivity at the national level between 2009/10 and 2013/14 and also considers specific subgroups of providers. The authors found that the group of small Trusts are more productive than larger ones, that there is variation between different regions and that broad relative (top or bottom half of the distribution) performance in level of productivity is generally stable over the period [5]. The Carter review also considered potential mechanisms for improving operational productivity, focusing particularly on procurement and found “unwarranted variations” in clinical and non-clinical resources, and in the quality and efficiency of the patient pathways [6].

Castelli et al. [7] examined variations in Trusts’ productivity in the English NHS for the years 2008/09 and 2009/10. They found extensive variations across Trusts, from between +33% above to -62% below the national average across the two years. Productivity was found to be lower in Trusts with greater financial autonomy, in Trusts receiving a high proportion of their income from education, research and development, and training activities and treating a greater proportions of both elderly and children patients.

This paper investigates the possible drivers of productivity variations in English Trusts over a period (2010/11–2012/13) characterised by important structural changes of the NHS and covering the initial period of the ‘Nicholson Challenge’ [2]. Following Castelli et al. [7], we use their cross-sectional measure of hospital productivity to analyse where these potential productivity differences may come from. Our work extends that of Castelli et al. [7] in that we broaden the definition of outputs to more fully reflect the whole array of diverse activity provided by hospitals. As noted in Appleby et al. [2], a key approach to improving productivity has been in moving the setting of care from overnight inpatient to day case, to outpatient to care in the community. We also consider additional factors with past evidence of affecting productivity, such as skill-mix of the hospital workforce [2] or higher resource use such as patients in their last year of life [8].

The structure of the paper is as follows. The productivity measures used in this work are defined in Section 2. This section also contains the specification of the regression model used and a description of the explanatory variables. Data used to populate the productivity measures and the explanatory variables are described in Section 3. Section 4 reports the results from the regression analyses. Discussion and concluding remarks are provided in Section 5.

## Methods

This was a retrospective analysis of previously collected, non-identifiable information, and involved no change in the management of patients. Obtaining individual consent was not feasible, so patient records are anonymized and de-identified prior to being shared with the research team. The Health and Social Care Information Centre (HSCIC) (now NHS Digital) handles requests for de-identified data and has legal responsibility to ensure there is an appropriate legal basis to permit the release and subsequent processing of data, that all necessary approvals are in place, and that organisations have appropriate arrangements and safeguards for secure data handling. The HSCIC approved the release of the Hospital Episode Statistics (HES) (content.digital.nhs.uk/hes) data to the University of York (DARS-NIC-03452-G8Z1V-v1).

Hospital Trusts are an administrative unit within the English National Health Service (NHS). Each Trust manages one or more hospitals and in some cases other sites where care is provided. Trusts are the unit for which we construct the productivity measures in this work. The aim of this study is to uncover factors that could potentially drive variation in Trusts’ productivity. Our dependent variable, *y*_*h*_, is a measure of productivity for each Trust in the English NHS. This is defined as the ratio of the total amount of outputs (patient treated and health care goods and services delivered) over total inputs (Labour (NHS staff and agency staff), Capital and Intermediate inputs) or labour inputs only (NHS staff and agency staff). Following Castelli et al. [7], the measure of Total Factor Productivity (TFP) for each hospital Trust *h* is calculated as:

$$y_{h}=P_{h}^{TF}=\frac{X_{h}}{Z_{h}^{TF}}=\frac{\sum_{j=1}^{J}x_{jh}{\bar{c}}_{j}}{\sum_{n=1}^{N}z_{nh}\omega_{n}+E_{h}^{A}+E_{h}^{M}+E_{h}^{K}}$$ (1)

where *X*_*h*_ is the total amount of outputs produced and $Z_{h}^{TF}$ is the total amount of all inputs used by Trust *h*.

We use physical measures of volume and price (cost) wherever possible and expenditure measures where physical ones are not available [9]. Trust’s outputs *x*_*jh*_, with *j* denoting type of output, are aggregated into an overall measure using national average unit costs ($\bar{c}$). Unlike Castelli et al. [7], we do not incorporate elements of health care quality in the calculation of the hospital output. Quality of health care is, however, considered in our analysis as one of the factors that could potentially explain variations in Trusts’ productivity. Inputs are calculated using a mixture of volume data on NHS hospital staff (*z*_*nh*_) weighted by the national average salary, *w*_*n*_, and expenditure data for agency staff, intermediate and capital inputs.

Finally, we standardise the productivity ratios (both TFP and Labour) for each Trust against the national average productivity ratio and convert them into a percentage term. This eases interpretation of the productivity ratios and comparisons across providers.

Variations in Trust productivity are then estimated by means of an Ordinary Least Squares (OLS) regression model with robust standard errors to account for potential heteroscedasticity. In the empirical literature on hospital / health system performance and health policy reform, a number of factors have been found to influence performance. These factors can be grouped into those pertaining to provider and workforce characteristics, patient characteristics, efficiency in resource use and health outcomes or quality of care. We have identified a number of factors which we included in our regression analysis. The OLS regression model takes the following form:

$$y_{h}=\beta_{0}+\sum_{g=1}^{5}\beta_{g}H_{gh}+\sum_{g=6}^{9}\beta_{g}P_{gh}+\sum_{g=10}^{11}\beta_{g}E_{gh}+{\beta_{12}Q}_{12h}+\varepsilon_{h}$$ (2)

where *H*_*gh*_ denotes those variables pertaining to the Trust and workforce characteristics, *P*_*gh*_ denotes patients characteristics, *E*_*gh*_ variables relating to the efficiency of Trusts and *Q*_*gh*_ to quality of care.

### Trust and workforce characteristics

In 2003, the Health and Social Care Act established NHS Foundation Trusts (FTs). The objective was to provide high performing Trusts with greater freedom, with Trusts tasked with translating this greater freedom into greater benefits to their patients [10]. Providers that qualified to become FTs were transformed into not-for-profit public organisations with greater managerial and financial autonomy from direct central government control [11]. FTs are allowed to keep surpluses, which they can use to either increase staff salaries and/or to re-invest in capital equipment. Further, FTs are allowed to borrow money (from various sources) to invest in improved services for patients and service users [12]. The first FTs were introduced in the English NHS in 2004/05, with the expectation that these should be more productive, introduce greater innovation and obtain greater on the job satisfaction [13, 14], given their new incentive structure. In our analysis, we control for FT *versus* non-FT status of the Trusts, with the *prior* expectation that the former should be more productive than their non-FT counterparts.

Street et al. [15] found that teaching activity introduces delays to the treatment process as part of a consultant’s role is to train medical students. Furthermore, teaching hospitals tend to treat more complex and/or more severe patients. Consequently, they are thought to have higher costs and thus appear less productive than non-teaching hospitals. In order to understand whether teaching activity is a potential driver of differences in Trusts’ productivity, we explicitly take this characteristic of a provider into account. We extend the more frequent practice of introducing a teaching dummy variable in regression analyses by identifying the extent of teaching activities provided in a Trust by measuring the total number of undergraduate medical students placed in any Trust. This variable more precisely captures levels of teaching activity across Trusts than a simple dichotomous variable.

Another Trust characteristic often linked to performance/efficiency is size. There are two *priors* in this respect acting in opposite directions: on the one hand, larger Trusts are thought to benefit from scale economies and to acquire experience from greater throughput; on the other, they might face diseconomies from greater complexity of organizational structure. Trust size has been measured in terms of either throughput or number of beds. Propper et al. [16] consider both measures of size in modelling Trust performance in terms of death rates, whilst Kolstad and Kowalski [17] and Aiken et al. [18] use only the number of beds to adjust for Trust size. Recognising that size is positively correlated with teaching activity, Teaching Trusts being also large general Trusts, including a measure of size enables us to disentangle scale effects from pure teaching effects. We use number of beds as our preferred measure of size in this paper, as this has the advantage of being independent from approaches to treatment which impact on throughput, such as the use of day cases. We consider this feature separately in the model.

One potential cause of inefficiency in a Trust is from bottlenecks in treatment caused by insufficient supply of specific skills. For example, a delay in the diagnosis and start of treatment if a specialist is not immediately available. It is noted in Appleby et al. [19] that roles within the NHS can be relatively rigid. This increases the occurrence of bottlenecks as it limits the potential for substitution between groups of staff. We capture one element of skill-mix through the percentage of medical workforce employed over total workforce employed by each Trust. The impact of a different skill mix on productivity depends on the relationship between the Trust’s chosen skill mix and its optimal skill mix. So while we do not have a specific prior for this variable, a positive correlation with productivity suggests that, on average, the supply of skills provided by doctors is a binding constraint on productivity.

Finally, the geographical location in which Trusts operate matters because there are unavoidable geographical differences in production costs. The Department of Health / Monitor account for these by adjusting local costs by the Market Forces Factor (MFF). The measure includes several elements of providers’ running costs for non-medical staff, medical and dental staff, land and buildings [20]. We expect these variables to be negatively related to the Trust productivity measures due to the presence of higher costs for the same level of output. We use the Overall MFF in the TFP regression models and the Staff MFF in the Labour Productivity regression models.

### Patient characteristics

The practice of prospectively allocating patients to a finite number of categories (often known as Diagnosis Related Groups, DRGs), an attempt to compare like with like patients in terms of cost, is common to many OECD countries. In England these groups are referred to as Healthcare Resource Groups (HRGs). We use the national average costs of HRGs to value output from the inpatient setting. HRGs do not capture all patient characteristics that can affect how resource intensive it is to treat them, e.g. age, but we give the same weight (national average cost) to all patients in a given HRG, irrespective of the actual cost to the Trusts treating them. This can translate into variation in the productivity measure as Trusts treating patients in a given HRG will have the same valued outputs but potentially have different amounts of inputs (costs) associated with that activity. We can adjust for potential systematic discrepancies by including patient characteristics known to affect cost in our regressions. If HRGs perfectly capture these characteristics, we would expect them to be insignificant determinants of our measure of productivity.

Castelli et al. [7], for example, controlled for the percentage of female patients and the percentage of patients falling into three age groups: aged 0 to 15 years, aged 46 to 60 years and over 60 years, with patients aged 16 to 45 years forming the reference category. Regarding age, it has generally been found that both older and younger patients are associated with higher costs. Also that the cost profile differs by gender, for example higher costs among women during child bearing years [21]. Further, it has been found that older patients tend to have multiple comorbidities and, as a consequence, that treating them is more resource and cost intensive [22]. Similarly, treating young and very young children, especially newborns, is more costly because they usually require specific specialized care [23].

In this study, we control only for age of patients treated, as defined in Castelli et al. [7].

Further, a recent report by the Health Foundation [8] found that the use of health care services (and hence the spend on these services) increases not only with age, but is considerably higher for people who are in their last year of life. This is known also as the ‘red herring’ hypothesis. In light of these findings, we control for the proportion of patients that are in their last year of life, and our *prior* is that hospital Trusts treating a higher proportion of patients in their last year of life bear higher costs and are consequently less productive. It has also been noted that time to death is ultimately a proxy for comorbidities [22]. We do not find chronic condition variables to be significant but this may reflect the small sample size inherent in considering Trust level variation instead of patient level variation.

### Efficiency in resource use

Hospital Trusts are increasingly asked to think of new and innovative ways of transforming service delivery to speed up care, improve care quality and patient experience, to the ultimate end of saving costs and increase efficiency. Ways of achieving this include “re-designing or shifting services away from the traditional setting of the hospital and out towards community based care” [24]. To this end, the Department of Health has developed the so-called ‘Better Care, Better Value’ indicators which summarise providers’ performance on a number of indicators and which can be used “locally to help inform planning, to inform views on the scale of potential efficiency savings in different aspects of care and to generate ideas on how to achieve these savings” [24]. We use two of the ‘Better Care, Better Value’ indicators as potential drivers of variation in Trusts productivity: length of stay and day surgery rates.

Trusts with shorter average length of stay and with a greater proportion of their elective activity carried out as day cases are expected to be more productive. Reduction of length of stay is one of the approaches noted in Appleby et al. [2].

### Quality of hospital care

In terms of quality of care we consider survival rates at Trust level. Mortality or, its mirror, survival rate is a simple measure of quality with the advantages of being clearly defined and straightforward to observe. As such, mortality remains a key measure of hospital performance. “Preventing people from dying prematurely” is one of five overarching measures used in the NHS Outcomes Framework 2011/12 [25] and one of the areas of assessment in the recent Keogh Review [26] of 14 specific Trusts.

We expect Trusts’ survival rates to be negatively related to Trusts’ productivity, both in terms of Labour and Total Factor, because providing better care to patients should require the use of more resources, for any given level of activity, and hence result in lower productivity.

We use only one indicator of hospital care quality, namely survival rate. This is due to the unavailability of robust and (time) consistent indicators (both in terms of processes and outcomes) of the quality characteristics of health care activity delivered outside the usual inpatient setting. Castelli et al. [27] in their national productivity measure of the English NHS use waiting times and survival rates adjusted by life years gained to quality adjust Trust inpatient output. We find that the same measures at the Trust level introduce too much noise in our productivity estimates. Furthermore, they are indicative of factors outside the Trusts’ direct control, and not necessarily reflecting the quality of care provided [28]. For example, life years gained, measured in terms of life expectancy, at the Trust level are more an indication of the socio-economic characteristics of the patient population served by a Trust than of the quality of care provided.

## Data

### NHS outputs and inputs

Trust inpatient activity is extracted from the Hospital Episode Statistics (HES) database [29]. HES comprises more than 15 million patient records in each financial year. We aggregate these observations of care under a single consultant (Finished Consultant Episodes, FCEs) to output units of continuous care in the same Trust (provider spell) using the latest methodology [30]. We assign a value to each output unit using unit cost data provided in the Reference Costs (RC) dataset, according to the Healthcare Resource Group (HRG) the output is mapped to. Around 89% of provider spells are made up of a single FCE. If a spell has multiple FCEs the value assigned to the spell is that of the most expensive HRG in the spell, but the HRG itself is that observed in the first FCE [31]. The national average cost of a patient spell for each HRG form the set of cost weights *c*_*j*_, seen in Eq (1).

Volume and cost information on all other provider goods and services are derived from the Reference Cost dataset [32–34]. Supporting information (S1 Table) contains the full list of the various Trust outputs considered in this study.

Information on Trusts’ volume of NHS staff is taken from the Electronic Staff Record (ESR), through the NHS iView workforce database (https://iview.ic.nhs.uk/), combined with Payroll and Human Resources systems from the NHS to derive national average earnings for each occupational group (in 2012/13 there were 585 separate groups). The data contain numbers of FTE staff employed in the NHS. Finally, the Trusts’ expenditure on agency staff, capital and intermediate inputs is derived from official financial returns: Annual Accounts for Foundation Trusts (FTs) and Trusts’ Financial Returns (2010/11 and 2011/12) or Financial Monitoring Accounts (2012/13) for Non-FTs. As expenditure on agency staff is no longer readily identifiable in the financial returns for 2012/13, we have used data provided by the Department of Health instead.

Five Trust mergers occurred over the period under investigation. In a few cases, merging Trusts continued to report both output and input data separately after the merger occurred. In these instances, we attributed any information on outputs and/or inputs reported to the merged Trust. And as a validity check, we compared total figures post-merger with equivalent data from previous years to verify these were on trend and to exclude any potential double counting. None was detected.

### Regressors

The explanatory variables included in our analyses come from various sources. These are set out in Table 1. Below we give further details of data preparation.

**Table 1:** Regressors–Description and source.

Variable	Description	Source
Number of Students (per 100 FTE)	$\frac{\mathrm{Number}\;\mathrm{of}\;\mathrm{students}}{\mathrm{Medical}\;\mathrm{workforce}\;+\;\mathrm{non-medical}\;\mathrm{workforce}}*\;100$	DH
Foundation Trust Indicator	Equal to one if Trust has FT status, zero otherwise	Monitor (1)
Size [number of beds]	Average number of total available beds	NHS England (2)
Medical / Workforce [%]	$\frac{\mathrm{Medical}\;\mathrm{workforce}}{\mathrm{Medical}\;\mathrm{workforce}\;+\;\mathrm{non-medical}\;\mathrm{workforce}}*\;100$	DH
Staff MFF [%]	Staff MFF * 100	DH
MFF [%]	Overall MFF * 100	DH
30-day Survival Rate [%]	$\left(1\text{-}\frac{\mathrm{Deaths}\;\mathrm{in-hospital}\;\mathrm{or}\;\mathrm{within}\;30\;\mathrm{days}\;\mathrm{of}\;\mathrm{discharge}}{\mathrm{Total}\;\mathrm{number}\;\mathrm{of}\;\mathrm{spells}}\right)*100$	Derived from HES and ONS
Patients in last year of life [%]	$\frac{\mathrm{Spells}\;\mathrm{with}\;\mathrm{patients}\;\mathrm{in}\;\mathrm{last}\;\mathrm{year}\;\mathrm{of}\;\mathrm{life}}{\mathrm{Total}\;\mathrm{number}\;\mathrm{of}\;\mathrm{spells}}*\;100$	Derived from HES and ONS
Patients aged 0–15 [%]	$\frac{\mathrm{Spells}\;\mathrm{with}\;\mathrm{patients}\;\mathrm{aged}\;0–15\;\mathrm{years}}{\mathrm{Total}\;\mathrm{number}\;\mathrm{of}\;\mathrm{spells}}*\;100$	Derived from HES
Patients aged 46–60 [%]	$\frac{\mathrm{Spells}\;\mathrm{with}\;\mathrm{patients}\;\mathrm{aged}\;46–60\;\mathrm{years}}{\mathrm{Total}\;\mathrm{number}\;\mathrm{of}\;\mathrm{spells}}*\;100$	Derived from HES
Patients aged over 60 [%]	$\frac{\mathrm{Spells}\;\mathrm{with}\;\mathrm{patients}\;\mathrm{aged}\;\mathrm{over}\;60\;\mathrm{years}}{\mathrm{Total}\;\mathrm{number}\;\mathrm{of}\;\mathrm{spells}}*\;100$	Derived from HES
Day Cases / Elective Spells [%]	$\frac{\mathrm{Day}\;\mathrm{cases}}{\mathrm{Number}\;\mathrm{of}\;\mathrm{elective}\;\mathrm{spells}}*\;100$	Derived from HES
Average LoS [days]	Average LoS (LoS = date spell ended—date spell started)	Derived from HES

Sources: DH = Department of Health; HES = Hospital Episode Statistics; ONS = Office for National Statistics.

Notes: (1) https://www.gov.uk/government/publications/nhs-foundation-trust-directory/nhs-foundation-trust-directory; (2) http://www.england.nhs.uk/statistics/statistical-work-areas/bed-availability-and-occupancy/bed-data-overnight/

The most complete dataset currently available for the number of full time medical undergraduate students is for the financial year 2011/12. Data for financial years 2010/11 and 2012/13 are not complete or directly comparable. In our analysis we therefore assume that the total number of full time students is stable over the three financial years considered. Where mergers occurred in 2012/13, the number of students in the constituent Trusts was summed to generate a figure for the merged Trust. Where mergers occurred in 2011/12, figures from merged Trusts in 2011/12 were apportioned to merging Trusts based on the ratio of the number of students reported by the Trusts in 2010/11.

The number of available beds is released quarterly by NHS England (http://www.england.nhs.uk/statistics/statistical-work-areas/bed-availability-and-occupancy/bed-data-overnight/). In order to make maximum use of this information, the average number of beds available in the four quarters of each financial year is used as our measure of size for each Trust. Some Trusts do not report the number of beds for every quarter; in this case, the average of the quarters where the number of beds is reported is used as the measure of size. Where Trusts merged within a financial year, beds information is available for the constituent Trusts of the merger for some quarters and the merged Trust for others; in these cases, the sum of beds available in constituent Trusts is taken as the number of beds available in the merged Trust for quarters before the merger.

Medical workforce comprises of doctors only, while non-medical workforce includes all other types of staff, e.g. nurses, midwives, ambulance staff, support staff. Data were provided by the Department of Health.

A patient is defined as being in their last year of life if his/her reported date of death occurs within one year of the start of the spell. This variable is calculated using the date of death data collated by the Office of National Statistics (ONS), which we merge to HES. From the same data, we identify deaths occurring within 30 days from discharge, from which we derive the 30 day survival variable.

From HES, we also construct the average length of stay measures for all Trust elective and non-elective patients, the proportion of day cases over total elective admissions and the four age groupings.

## Results

Summary statistics of all Trusts’ activity provided in the different health care settings and inputs for the years 2010/11 to 2012/13 are reported in Table 2. The total number of Trusts varies by year, with 166 in 2010/11, 164 in 2011/12 and 161 in 2012/13. Not all Trusts provide activity in all the settings; hence, the variation in the total number of providers reporting activity in each setting. In particular, all Trusts provide both inpatient and outpatient activity; less than 30 Trusts in our sample provide any activity related to Community Mental Health, with the number of Trusts providing activity in any of the remaining sectors varying between these two extremes. Finally, we note that two providers did not report Direct Labour data in 2010/11, and thus were excluded from the analyses for that year.

**Table 2:** Summary statistics for NHS outputs and inputs, 2010/11–2012/13.

Variable		2010/11			2011/12			2012/13	
	N	Mean	Std. Dev.	N	Mean	Std. Dev.	N	Mean	Std. Dev.
**Hospital Outputs**									
**Elective and day cases**	166	41,966	24,254	164	43,561	24,648	161	44,691	25,292
**Non-Electives**	166	43,452	24,681	164	43,835	24,089	161	44,891	25,203
**A&E**	152	99,993	46,158	151	109,302	51,385	148	112,390	57,084
**Chemo/Radiotherapy & High Cost Drugs**	164	31,807	39,502	161	31,299	36,340	160	42,014	52,257
**Community Care**	149	76,270	121,177	147	221,778	298,557	144	238,663	310,998
**Community Mental Health**	24	11,344	11,390	27	61,229	169,587	28	111,964	258,957
**Diagnostic Tests**	150	2,119,259	1,315,580	154	2,176,772	1,433,650	152	2,234,692	1,503,636
**Hospital/Patient Transport Scheme**	84	4,986	5,193	N/A			N/A		
**Other NHS Activity**	154	24,425	15,737	153	27,664	17,037	152	28,101	17,343
**Outpatient**	166	435,269	227,969	164	437,076	228,661	161	451,489	249,937
**Radiology**	165	50,148	31,421	162	53,370	32,095	160	58,155	38,833
**Rehabilitation**	86	15,213	12,906	96	18,132	17,330	93	16,813	17,137
**Renal Dialysis**	67	59,149	51,470	61	66,355	49,342	64	64,624	53,073
**Specialist Services**	163	20,259	15,319	161	23,612	18,160	158	26,727	21,312
**Hospital Inputs (£000)**									
**NHS Labour (Direct)**	164	137,584	84,297	164	145,049	84,385	161	153,940	90,913
**Agency Labour**	166	7,672	6,335	164	7,415	5,581	161	9,615	7,446
**Intermediate goods and services**	166	65,239	48,405	164	73,453	53,398	161	102,285	84,558
**Capital**	166	32,541	23,783	164	38,781	28,761	161	62,497	52,005

Summary statistics for the variables used in the regression analyses are set out in Table 3.

**Table 3:** Summary statistics explanatory variables, 2010/11–2012/13.

Variable	2010/11			2011/12			2012/13		
	N	Mean	Std. Dev.	N	Mean	Std. Dev.	N	Mean	Std. Dev.
**Number of Students (Per 100 FTE)**	166	2.07	1.50	164	1.96	1.41	159	1.91	1.38
**Foundation Trust Indicator**	166	0.56	0.50	164	0.57	0.50	161	0.61	0.49
**Size [Number of Beds]**	164	670.56	362.89	164	678.09	378.77	161	681.93	374.97
**Medical / Workforce [%]**	166	12.71	2.23	164	12.41	2.38	159	12.43	2.44
**30 Day Survival Rate [%]**	166	97.47	0.91	164	98.64	0.47	161	98.59	0.48
**Patient in last year of life [%]**	166	8.71	4.20	164	8.87	4.15	161	9.00	4.08
**Patient aged 0–15 [%]**	166	14.47	13.68	164	14.29	13.70	161	14.42	13.87
**Patient aged 46–60 [%]**	166	16.54	4.45	164	17.02	4.24	161	17.22	4.28
**Patient aged over 60 [%]**	166	39.69	10.44	164	40.29	10.69	161	40.75	10.67
**Day Cases / Elective Spells [%]**	166	75.75	10.93	164	77.07	10.30	161	77.85	10.81
**Average LoS [Days]**	166	2.71	0.57	164	2.65	0.57	161	2.33	0.58
**Staff MFF [%]**	166	100.51	9.93	164	100.47	9.98	N/A		
**Overall MFF [%]**	166	100.70	6.71	164	100.68	6.75	N/A		

Note: In 2010/11, the variable ‘size’ is missing for Rotherham NHS Foundation Trust (RFR) and Sheffield Teaching Hospitals NHS Foundation Trust (RHQ). In 2012/13, Isle of Wight NHS Trust (R1F) and Barts Health NHS Trust (R1H) did not report non-medical workforce information; therefore, we were not able to calculate the percentage of medical workforce over total workforce and the ‘Number of Students per 100 FTE’ variables.

Please note that the number of medical students is assumed to be time invariant; hence, variation in the proportion of students per 100 FTE staff reflects changes over time in total workforce within a Trust as well as organisational changes (mergers). The number of students to workforce ratio is around 2%. A small number of Trusts acquires FT status during the study period, the proportion of Trusts with FT status increases from 0.56 to 0.61. The average Trust contains 671–682 beds, employs 12% of medical staff and has an average survival rate of 98%. Around 9% of patients treated are in their last year of life. There are improvements in terms of day case rate (increasing) and length of stay (decreasing) over time.

The results for the Ordinary Least Squares (OLS) models of Labour (LP) and Total Factor Productivity (TFP) are presented in Table 4.

**Table 4:** OLS cross-section models of hospital productivity scores, 2010/11–2012/13.

	Labour Productivity						Total Factor Productivity					
	2010/11		2011/12		2012/13		2010/11		2011/12		2012/13	
**Number of Students (per 100 FTE)**	0.706		-0.110		0.074		0.205		-0.818		-0.473	
	(1.003)		(0.826)		(0.761)		(0.793)		(0.733)		(0.841)	
**Foundation Trust Indicator**	1.549		-0.783		-1.133		-2.080		-2.669	*	-9.461	***
	(1.949)		(2.077)		(1.752)		(1.689)		(1.594)		(1.717)	
**Size [number of beds]**	-0.008	**	-0.005	*	-0.003		-0.009	***	-0.007	***	-0.006	**
	(0.003)		(0.003)		(0.003)		(0.002)		(0.002)		(0.003)	
**Medical / Workforce [%]**	0.886		1.024		0.948	*	-0.035		0.009		-1.182	**
	(1.292)		(0.781)		(0.502)		(1.005)		(0.602)		(0.513)	
**MFF [%] (1)**	-0.300		-0.341	**	-		-0.195		-0.727	***	-	
	(0.251)		(0.164)				(0.287)		(0.189)			
**30-day Survival Rate [%]**	0.596		3.571		4.330		-6.587	**	-5.822		-6.938	*
	(3.578)		(5.487)		(4.265)		(3.088)		(4.658)		(3.976)	
**Patients in last year of life [%]**	1.091		1.046	**	1.453	**	-0.494		0.203		0.411	
	(1.092)		(0.526)		(0.603)		(0.807)		(0.432)		(0.531)	
**Patients aged 0-15 [%]**	-0.646	***	-0.717	***	-0.817	***	-0.548	***	-0.704	***	-0.888	***
	(0.223)		(0.270)		(0.229)		(0.168)		(0.249)		(0.214)	
**Patients aged 46-60 [%]**	-1.425	*	-2.098	**	-2.204	***	-1.535	***	-2.092	***	-2.693	***
	(0.742)		(0.860)		(0.658)		(0.576)		(0.775)		(0.615)	
**Patients aged over 60 [%]**	-0.023		0.050		-0.115		-0.070		-0.102		-0.166	
	(0.230)		(0.230)		(0.216)		(0.174)		(0.216)		(0.199)	
**Day Cases / Elective Spells [%]**	-0.247		-0.209		-0.340	**	0.076		-0.014		-0.143	
	(0.181)		(0.166)		(0.136)		(0.140)		(0.158)		(0.140)	
**Average LoS [days]**	-1.379		1.821		-3.188		-4.968	*	-3.548		-7.986	***
	(3.368)		(3.615)		(2.473)		(2.765)		(3.141)		(2.902)	
**N**	162		164		159		162		164		159	
**R-Squared**	0.2696		0.2648		0.3372		0.4327		0.5156		0.5511	

All regressions include a constant, not reported in the Table.

***, ** and * indicate 1%, 5% and 10% significance, respectively. Robust standard errors in parentheses.

(1) Staff MFF in Labour Productivity regressions, and Overall MFF in TFP regressions.

Foundation Trusts are found to be not statistically significantly different from non-FTs when it comes to their LP measure, and perform worse than non-FTs when the TFP measure is considered in both 2011/12 and 2012/13.

We find a small negative association between both LP and TFP and Trust size, though more strongly significant for the TFP measure.

A positive association between LP and the proportion of medical workforce is consistent across time but only significant in the last financial year of our analysis, and then only at 10% level. The association is negative for the TFP measure.

We find a negative association (significant only in 2011/12) between both LP and TFP and the Market Forces Factor.

Regarding the variables measuring patients’ characteristics, we find a positive association between patients in their last year of life and LP, and that hospital Trusts treating a relatively higher proportion of patients in age groups 0–15 and 46–60 are less productive compared to those treating a higher proportion of patients in the reference group (16–45 years).

We find a consistently negative association between both measures of productivity and the proportion of elective activity performed as day cases. This result is, however, only significant in the LP model for 2012/13.

Trusts that keep their patients in hospital for longer periods of time have on average lower productivity, whichever measure of productivity is considered, albeit this association is found to be statistically significant only for the TFP measure.

Our results show that 30 day post discharge survival rate is associated with lower TFP for the financial years 2010/11 and 2012/13. The coefficient is of similar size for 2011/12, albeit not significant.

## Discussion

This paper examines variations in Trusts’ productivity with the aim to uncover potential drivers of said variations for English Trusts. Similarly to Castelli et al. [7], we find Foundation Trusts to be less productive than non-Foundation Trusts. Castelli et al. [7] also noted that the difference between FTs and non-FTs disappeared if Labour Productivity was considered, concluding that the capacity for FTs to make capital investments may be reflected in lower productivity in the short term and that the additional capital investment had not “yet yielded a proportionate increase in output” [7]. The continued presence of a difference between the two measures of productivity considered in our paper may indicate that FT investment in capital has continued in subsequent years and this in part offsets productivity benefits of earlier investments.

Surprisingly, treating more patients in their last year of life is associated with higher Labour Productivity. The counter-intuitive result might be due to the fact that the proportion of patients in their last year of life is more closely linked to inpatient activity, rather than the diverse array of healthcare goods and services considered in this analysis. Inpatient activity represents between 49% and 51% of the total value of all Trust activity in the financial years considered here. So, as a sensitivity analysis, we restricted output to inpatient activity only, finding a strong and negative association between patients in their last year of life and both measures (Labour and Total Factor) of Trusts’ productivity.

The relation between Trusts’ size and productivity seems to support the idea that diseconomies of scale faced by larger Trusts, due to their more complex organisational structure, dominate the economies of scale enjoyed by these providers of higher throughput and reduced procurement costs. Our finding of higher productivity among smaller Trusts concurs with that of the Health Foundation Report [5].

The positive association between the proportion of medical workforce (over total workforce) and Labour productivity may indicate that medical staff is an important component of the skill mix of more productive Trusts.

The geographical location in which a Trust operates seems to explain part of the variation found in Hospital Trusts’ productivity, both in terms of Labour only and Total Factor Productivity, with a negative association found between the Market Forces Factor and both measures of productivity analysed: an indication that higher costs for either labour only or all inputs are indeed reflected in lower productivity, as would be expected.

The negative association found between survival rate and Trusts productivity might be an indication that higher quality requires greater resources, in terms of increased use of inputs per patient. This is particularly true for the Total Factor Productivity measure, which may indicate that Trusts are investing in technology that improves the quality of health outcomes rather than simply increasing throughput. Another potential mechanism is that survival, all else being equal, results in further costs for the same value of output, in a similar way to longer length of stay.

Finally, we are not able to explain why Trusts treating a greater proportion of patients as a day case are less productive. To gain further understanding of this result, we have run a number of sensitivity tests, using alternative definitions of the day case variable, including an activity weighted version, but still obtain similar results.

A limitation of our study is that patient characteristics are limited to the inpatient setting. Inpatient information from HES is very rich but characteristics of patients receiving care in other settings are not observed. To draw conclusions about more general activity we must assume the characteristics of inpatients are a reasonable proxy for the characteristics of patients receiving care in other settings. However, we note that the inpatient setting is the dominant one in terms of *monetary value* of activity provided in the NHS.

Another constraint in our analysis for drivers of variation is the relatively small sample of observations. This limits the complexity and variety of mechanisms which can be assessed. We therefore limit explanatory variables to those with evidence of past utilization and effect in previous literature and focus on the direction and significance of these rather than specific magnitudes.

Despite the above limitations, our study adds to the current knowledge by extending the definition of hospital output used in previous analyses of hospital variations / efficiency [5, 7] to include all health care goods and services produced and delivered by hospitals. Castelli et al. [7] and Lafond et al. [5] limit their analysis to inpatient and outpatient activity and to inpatient and accident and emergency activity, respectively.

We also consider both the Total Factor and Labour Productivity measures separately in an attempt to understand whether variations in these two measures are driven by different factors.

Finally, our study considers new drivers of potential variation in Trusts’ productivity by controlling for a measure of skill-mix of hospital staff (a similar measure was included in the analysis by Lafond et al. [5]), unavoidable geographic differences in production costs through the use of the MFF, controlling for the proportion of patients treated who are in their last year of life and finally, controlling for the proportion of elective activity carried out as day cases. All of the above factors considered in our analysis were mentioned in the Nicholson challenge as the areas that should drive the £20 billion savings.

## Supporting information

S1 Table Hospital settings, description of outputs and unit of measurement. (DOCX)
