# Supplementary material for: Hospital Trusts productivity in the English NHS: Uncovering possible drivers of productivity variations
Source: PLoS One. 2017 Aug 2;12(8):e0182253. doi: 10.1371/journal.pone.0182253 (PMC5540600; doi:10.1371/journal.pone.0182253)
Supplement: S1 Table — (DOCX) [file pone.0182253.s001.docx]

# S1 Table

S1 Table Hospital settings, description of outputs and unit of measurement

| **Hospital settings** | **Description of outputs** | **Unit Type** |
| --- | --- | --- |
| A&E Services, incl. Ambulance services | Emergency Department, Minor Injury Units, Walk-in-Centres, Specialised Emergency (non-24 hour) Department | Attendances (leading or not leading to Admitted Patient Care); Call, Patient, Incidence |
|  |  |  |
| Chemo/Radiotherapy & High Cost Drugs | Chemotherapy and Radiotherapy sessions, High Cost Drugs | Treatment cycles, Deliveries, Attendances, Fractions, Spells |
|  |  |  |
| Community Care | District nursing and health visitor services for routine and specialist services outside hospitals (e.g. patients’ homes, local health centres, etc.), also services provided in local areas in the wider community (including hospital bases if necessary) such as midwifery, podiatry, speech therapy etc. | Contacts, HRG codes, Attendances, Visits, Vaccinations |
|  |  |  |
| Community Mental Health | Children and adolescent mental health services, drug and alcohol services, specialist mental health services (e.g. autistic spectrum disorder and eating disorder services) and secure mental health services. From 2011/12 also mental health care clusters for working age adults and older people reporting service user needs over extended periods of time (min 4 wks to 1 year) | Bed Days, Assessments, Cluster Days, Patient days, Contacts, Attendance |
|  |  |  |
| Diagnostic Tests | Direct Access Diagnostic and Pathology Services undertaken in admitted patient care, critical care, outpatients or emergency medicine | Tests |
|  |  |  |
| Hospital/Patient Transport Scheme | Financial assistance to NHS patients who who require assistance in meeting the cost of travel to and from their care | Attendance, divided by Admitted Patient Care, Outpatient and Other |
|  |  |  |
| Inpatient | Elective, day cases and non-electives (emergency and maternity admissions) | HRG codes |
|  |  |  |
| Other NHS activity | Audiological Services, Hospital at home (until 2011/12), Day Care Facilties, Regular Day and Night Admissions | Attendances, Aids issued, Screenings, Contacts, Repairing, Patient Days, Admissions, |
|  |  |  |
| Outpatient | Consultant and Non-Consultant led visits held at clinics in hospitals, community health centres, general practices or other locations. Outpatient activity with procedures are reported separately | Procedures, Attendance (Face/non Face to Face, Single/multi professional, First attendance/Follow Up), HRG codes |
|  |  |  |
| Radiology | Diagnostic Imaging | Examinations |
|  |  |  |
| Rehabilitation | Rehabilitation Services, | Bed Days, Attendance |
|  |  |  |
| Renal Dialysis | Renal dialysis, covering both renal and peritoneal dialysis | Sessions |
|  |  |  |
| Specialist Services | Specialist Palliative Care, Cystic Fibrosis, Critical Care Services, Coronary Care Unit (only in 2010/11), Cancer Multi Disciplinary Teams | Bed Days, Attendances, Patient Journey, Outreach Services, Patients, Treatment Plan |
